# Do health assessments affect time to permanent residential aged care admission for older women with and without dementia?

**DOI:** 10.1111/ggi.14631

**Published:** 2023-06-29

**Authors:** Julie Byles, Dominic Cavenagh, Jamie Bryant, Mariko Carey, Danielle Mazza, Rob Sanson‐Fisher

**Affiliations:** ^1^ Centre for Women's Health Research University of Newcastle Newcastle New South Wales Australia; ^2^ Women's Health Research Program Hunter Medical Research Institute New Lambton Heights New South Wales Australia; ^3^ School of Medicine and Public Health University of Newcastle Newcastle New South Wales Australia; ^4^ Equity in Health and Wellbeing Program Hunter Medical Research Institute New Lambton Heights New South Wales Australia; ^5^ Department of General Practice Monash University Melbourne Victoria Australia; ^6^ Health Behaviour Research Collaborative University of Newcastle Newcastle New South Wales Australia; ^7^ Hunter Medical Research Institute University of Newcastle Newcastle New South Wales Australia

**Keywords:** health assessments, health service use, residential aged care

## Abstract

**Aim:**

To investigate the effect of health assessments on permanent residential aged care admission for older Australian women with and without dementia.

**Methods:**

A total of 1427 older Australian women who had a health assessment between March 2002 and December 2013 were matched with 1427 women who did not have a health assessment in the same period. Linked administrative datasets were used to identify health assessment use, admission to permanent residential aged care, and dementia status. Outcome was time to residential aged care admission from the matched date of health assessment.

**Results:**

Women who had health assessments were less likely to be admitted to residential aged care in the short term (100 days), irrespective of dementia status (subdistribution hazard ratio [SDHR] = 0.35, 95% CI = [0.21, 0.59] for women with dementia; SDHR = 0.39, 95% CI = [0.25, 0.61] for women without dementia). However, there were no significant differences at 500‐ and 1000‐days follow‐up. At 2000‐days follow‐up, women who had a health assessment were more likely to be admitted to residential aged care, regardless of dementia status (SDHR = 1.41, 95% CI = [1.12, 1.79] for women with dementia; SDHR = 1.55, 95% CI = [1.32, 1.82] for women without dementia).

**Conclusions:**

Benefits from health assessments may depend on the recency of the assessment, with women less likely to be admitted to residential aged care in the short term after a health assessment. Our results add to a growing body of literature suggesting that health assessments may provide benefits to older people, including those with dementia. **Geriatr Gerontol Int 2023; 23: 595–602**.

## Graphical abstract

Older Australian women were less likely to be admitted to a residential aged care facility in the short term after receiving a comprehensive health assessment from a medical practitioner. There was no evidence to suggest that this association differed for women with and without dementia.

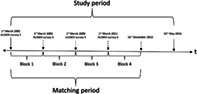

## Introduction

Health assessments for older people aged 75 years or older were introduced into the Australian health care system in 1999. These assessments are subsidized by the Medicare Benefits Schedule (MBS) and allow in‐depth assessment of an older person's medical, physical, psychological, and social functioning. The assessment can be carried out by a primary care doctor, or by a practice nurse under a doctor's supervision. The aim of the assessment is to identify complex problems in general health, functional abilities, mental health and social support, and to initiate appropriate preventive and educational strategies to improve health and prevent functional decline. Specific aspects of health assessments include assessment of blood pressure, pulse rate and rhythm, medications, continence, immunization status, activities of daily living, falls, cognition, hearing and vision, mood, and social support.^1^ Assessments are often initiated by the primary care provider.

Uptake of health assessments across the older population at introduction was rapid. In the 35 months following their introduction, an estimated 31% of all eligible older Australians had at least one Medicare claim for a health assessment, with women more likely to receive an assessment than men.^2^ Evaluation of the uptake of health assessments by women in the Australian Longitudinal Study of Women's Health (ALSWH) showed a steady increase from November 1999, with 49% of eligible women having an assessment by October 2003,^3^ and 61.8% having an assessment by 2013.^4^ Although people over 75 years of age are eligible to have an assessment every 12 months, most women who had any assessment only had one or two between 1999 and 2013.

Several studies have evaluated various modes of health assessments for older people living in the community.^5^, ^6^ While these studies show mixed results, there is an emerging consensus that geriatric health assessment can slow decline in physical function and possibly reduce admissions to aged care.^7^ However, in a randomized controlled trial of health assessments in Australia, health assessments were associated with a statistically significant increase in admissions to nursing homes.^6^ The authors attributed this possible increase to the identification of high‐level needs that could be best met in residential care and to facilitated access to care.

Dementia is a common progressive condition that affects cognition and functioning,^8^ and it is commonly comorbid with other chronic conditions in later life.^9^ In the earlier stages of the disease, most people with dementia live in the community (70%). However, many people with dementia will enter residential aged care (RAC) in their last months or years of life,^10^ and just over half of the individuals in RAC have dementia.^11^ RAC in Australia is a government‐subsidized program that provides permanent accommodation and personal care for older Australians who are no longer able to live in the community.^12^ Older people with dementia are at higher risk of admission to RAC compared with older people without dementia.^13^, ^14^ Health assessments may offer an opportunity for general practitioners to gain a more comprehensive overview of the needs of people with dementia and inform developing care plans. A recent analysis of the use of health care by women with and without dementia^15^ found that women with dementia had more GP attendances and fewer specialist attendances but were not more likely to receive health assessment, chronic disease management, or allied health services. Health assessments may be of particular value for people with dementia who have multiple complex health and social care needs.

In this study, we examine whether women who had a health assessment were more or less likely to be admitted to RAC and analyse the influence of dementia on this association.

## Methods

### Sample

**Figure 1:**
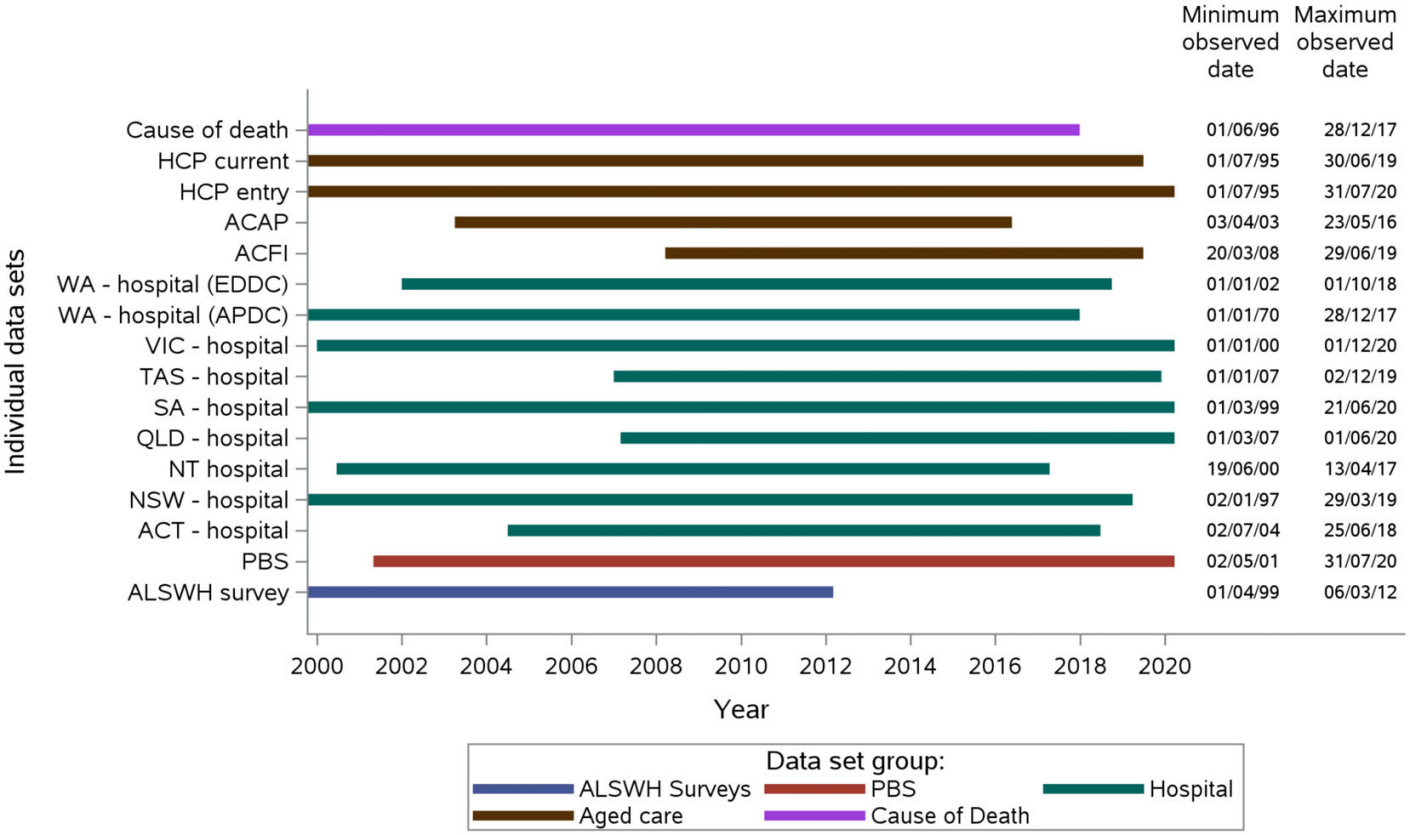
Date range for the datasets used for dementia identification.

**Figure 2:**
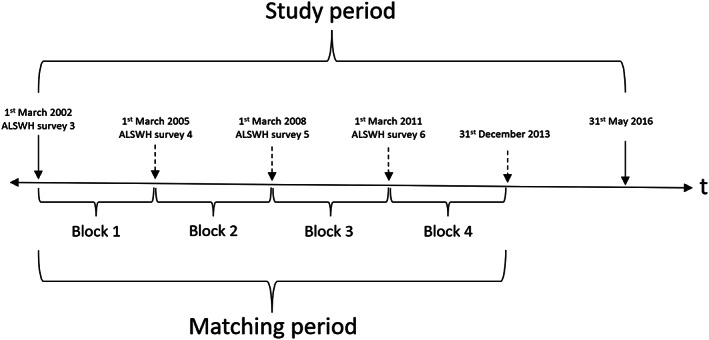
Timeline of the study period and blocks used for matching participants.

This study includes women from the 1921 to 1926 birth cohort of the ALSWH.^16^ Participants’ survey data were linked to data from the MBS, Pharmaceutical Benefits Scheme (PBS), State Hospital Admitted Patients Data Collections (APDC – all states and territories), Western Australia emergency data collection, Aged Care Assessment Program (ACAP), Aged Care Funding Instrument (ACFI), RAC admissions, and National Death Index (NDI). While the periods covered by each dataset are slightly different, all start on or before 2004, except for Tasmania and Queensland APDC (which both begin in 2007), and the ACFI dataset, which begins in 2008 (when the program was introduced). The ACAP dataset ends in May 2016, which is the earliest dataset end date (Fig. 1). Based on the date range of the linked administrative datasets, a study period of 1st March 2002 (beginning of ALSWH survey 3 administration) until 31st May 2016 (end of ACAP data coverage) was defined. The four ALSWH surveys during this period were used to define the beginnings of four time‐blocks in 2002, 2005, 2008 and 2011 (Fig. 2), with 2002–2013 being the period of exposure (i.e., the time during which the intervention occurring resulted in potential inclusion in the matching), and the rest of the time till the end of May 2016 serving as follow‐up time to observe the outcome(s) of interest. Of the 12 432 women of the 1921–1926 ALSWH birth cohort, women were eligible to be analysed if they had completed at least one ALSWH survey administered in 2002, 2005, 2008 or 2011, were living in the community at the time of their first health assessment, and consented to data linkage (see Figure S1).

### Measures

#### Intervention

Dates of health assessments (Medicare item numbers^17^ [unique service identifiers] 700–719 – “Health assessment for people aged 75 years and older”) were taken from the MBS data and used to classify people as having had a health assessment by 31st May 2016 (end of linked data coverage – see Fig. 1) or as never having had a health assessment before then.

### Outcome

Dates of admission to permanent RAC were taken from the aged care data.

### Covariate of interest

Dementia was identified using the methods described by Waller et al.^18^ from indicators in the ALSWH survey, PBS, state hospital, Aged Care and NDI datasets. Dementia indication from the ALSWH surveys was self‐reported responses to the question “In the last 3 years have you been diagnosed with or treated for: Alzheimer's Disease or Dementia” (this question was asked at surveys 2 [1999], 3 [2002], 4 [2005], 5 [2008] and 6 [2011]). PBS dementia indication was the supply of any of the following anti‐dementia drugs: Tacrine, Donepezil, Rivastigmine, Galantamine or Memantine. Hospital dementia indication was admission to hospital with any ICD10 (F00, F01, F03, G30) or ICD9 (290, 294.2, 331.0) dementia codes as a primary or secondary diagnosis. Aged Care dementia indication was an ACAP or ACFI record with a health condition listed as 50*, 51* or 532. NDI dementia indication was a cause of death ICD10 or nine code related to dementia (same codes as hospital data). Six months prior to the first indication of dementia (in any of these data sources) was used as a conservative proxy date for dementia diagnosis.^19^

### Other covariates

Health‐related measures taken from the ALSWH surveys in 2002, 2005, 2008 and 2011 included: self‐rated general health, classified as good (excellent, very good, good) and poor (fair, poor), SF36 mental health, general health, and physical functioning subscale scores,^20^, ^21^ and any fall to the ground in the last 12 months. The number of self‐reported chronic diseases ever reported was calculated at each survey using responses to questions on ever being diagnosed/treated for diabetes, heart disease, hypertension, osteoporosis, cancer, and arthritis. The number of chronic diseases was categorized into none or one, two or three, or four or more. MBS data were used to calculate the number of GP attendances (broad terms of service codes 0101, 0102, 0103) in the year before survey completion.

Demographic characteristics from the surveys included whether women had a concession card for health services (either a Commonwealth Seniors Health Card^22^ or Pensioner Concession Card^23^ – these are Australian government‐issued cards allowing their bearer access to cheaper health care, medicines and other discounts), their ability to manage on their available income (easy/not too bad or difficult/impossible), whether they were currently partnered (married/de facto vs. separated/divorced/widowed/single), and their ARIA+ area of residence (major city, inner regional, outer regional/remote/very remote).^24^ Participants’ highest educational qualification at Survey 1 was based on the survey question “What is the highest qualification you have completed” and was categorized as no formal qualifications, school qualifications only (School or Intermediate Certificate, Higher School or Leaving Certificate), or a tertiary qualification (trade/apprenticeship, certificate/diploma, university degree, higher university degree). Age was calculated from date of birth to survey completion.

### Statistical analysis

Logistic regression was used to calculate the individual probability of a woman having a health assessment in each time–block, given they had completed the survey at the beginning of the time–block. This probability was used as the basis for a propensity score matching approach to estimate a quasi‐experimental effect of health assessments.^25^ Women having their first health assessment in the study period during a block were matched with women who did not have a health assessment ever. Once women were matched, they could not be matched again in a later block. Measures used in the logistic regression propensity score calculation were age, SF36 general health, mental health and physical functioning scores, number of GP attendances, falls, concession card status, self‐reported general health, area of residence, partnered status, highest qualification, ability to manage on available income, and number of chronic diseases. Measures chosen were informed by previous research on the uptake of health assessments amongst this cohort of women.^3^, ^4^, ^15^ Multiple imputation using fully conditional specification^26^ with 25 imputations was used to account for missing data. Propensity scores were calculated for each imputation and then averaged across imputations for a final score. A 0.2 standard deviation calliper distance was used.^27^ Women who did not have a health assessment must have been alive for the entire month in which their match had the health assessment. Matched participant pairs were dropped if either of them was already in RAC on the date of the assessment. The group of matched women formed the analysis sample.

The number of matches per block was calculated. Standardized differences were calculated for each measure used in the propensity score calculation to assess group balance.^28^

A time to event approach was used to investigate the effect of health assessments on admission to RAC. The start of follow‐up was defined as the date of the first health assessment or their match's date of assessment (for those who did not have an assessment). The end point of interest was the date of admission to permanent RAC. A competing risk of death was also defined. If participants had not been admitted to RAC or died before May 31st 2016 then they were censored on this date. Health assessment and dementia were the two primary predictors of interest. Dementia was treated as a time‐varying covariate. Baseline covariates were taken from the survey before the start of follow‐up. Frequencies (categorical variables) or means and standard deviations (continuous variables) were calculated for all covariates by outcome event. Chi‐square and Kruskal–Wallis tests were used to test differences across outcomes. Median and first/third quartile follow‐up times were calculated. Fine and Grey competing risk models^29^ were fitted firstly with only the predictors of interest (health assessment, dementia, and their interaction), then with adjustment for the other covariates (age, number of chronic conditions, concession card status, fall in the last 12 months, self‐rated general health, SF36 general health score, number of GP visits in the last 12 months, SF36 mental health score, highest qualification, area of residence, ability to manage on available income, SF36 physical functioning score, partnered status). All model variables were checked for violation of the proportional hazards assumption by including an interaction of log time and the variable in the model. Interaction terms where *P* < 0.1 were retained in the final models to account for non‐proportionality. If a variable violated this assumption, outcomes were calculated at 100‐, 500‐, 1000‐ and 2000‐days follow‐up for that variable. Subdistribution hazard ratios (SDHRs) and their 95% confidence intervals (CIs) were calculated for all covariates. A 5% significance level was defined. All analysis was conducted in SAS 9.4 M7 (SAS Institute Inc., Cary NC, USA).

## Results

### Propensity score matching

There were 8728 women eligible for analysis, of which 5787 had their first health assessment after 2002 during one of the four time‐blocks, 1544 women had never had a health assessment, and 1397 were excluded owing to having their first health assessment outside the time‐blocks. During the matching period, 2956 women (1427 pairs of health assessment users/non‐users) were matched (2854 in block 1, 84 in block 2, 16 in block 3, and 2 in block 4). These women formed the analysis sample (see Figure S1). Logistic regression models calculating propensity scores had an average c‐statistic of 0.62 (std dev. = 0.004) across imputations, indicating average model prediction. All propensity score calculation variables had standardized differences less than 0.1 in all imputations, indicating good balance between the health assessment users/non‐users^28^ (see Table S2).

### Time to RAC

By the end of the analysis period, 1144 (38.7%) women had been admitted to permanent RAC, 1195 (40.43%) had died before admission to RAC, and 617 (20.87%) had experienced neither event (censored). There were statistically significant differences across all covariates by event type, except for area of residence, partnered status, highest qualification, ability to manage on available income, and concession card status (note: this variable had a high proportion of missing values) (Table 1).

**Table 1:** Baseline covariate means and frequencies across outcome event

	Censored (no event)		Death		Admission to RAC	
Continuous covariates	Mean	Std Dev.	Mean	Std Dev.	Mean	Std Dev.
Age*	78.05	1.67	78.45	1.53	78.66	1.62
SF36 mental health subscale score*	81.3	14.53	78.83	15.9	77.63	16.04
SF36 general health subscale score*	70.69	18.04	60.81	22.49	61.28	21.22
SF36 physical functioning subscale score*	67.44	24.75	50.89	29.69	51.37	28.66
Number of GP attendances in previous year*	7.86	5.68	10.84	9.16	10.96	8.12

Categorical covariates	%	*n*	%	*n*	%	*n*
75+ Health assessment*						
No	35.01	216	67.2	803	40.12	459
Yes	64.99	401	32.8	392	59.88	685
Dementia by start of follow‐up^a^,*						
No	99.00^c^	610^a^	98.24	1174	97.29	1113
Yes	<1^c^	<5^a^	1.76	21	2.71	31
Dementia by end of follow‐up^a^,*						
No	89.79	554	86.53	1034	54.63	625
Yes	10.21	63	13.47	161	45.37	519
Fall to the ground in the last 12 months*						
Missing	5.02	31	6.69	80	6.03	69
No	78.12	482	75.4	901	71.42	817
Yes	16.86	104	17.91	214	22.55	258
Concession card status						
Missing	10.21	63	36.57	437	28.15	322
No	33.71	208	22.85	273	25.79	295
Yes	56.08	346	40.59	485	46.07	527
Self‐reported general health*						
Missing	<1^c^	<5^b^	0.92	11	0.87	10
Excellent/good	81.00^c^	500^b^	62.01	741	63.46	726
Fair/poor	18.00^c^	110^b^	37.07	443	35.66	408
Area of residence						
Missing	<1^c^	<5^b^	<1^c^	<5^b^	<1^c^	<5^b^
Major city	44.00^c^	270^b^	41^c^	490^b^	40.00^c^	460^b^
Inner regional	32.00^c^	200^b^	36.00^c^	430^b^	39.00^c^	440^b^
Outer regional/rural/remote	23.00^c^	140^b^	22.00^c^	270^b^	20.00^c^	230^b^
Partnered status						
Missing	<1^c^	<5^b^	<1^c^	<5^b^	0.61	7
Partnered	44.00^c^	270^b^	39.00^c^	470^b^	39.86	456
Unpartnered	55.00^c^	340^b^	59.00^c^	710^b^	59.53	681
Highest qualification						
Missing	5.67	35	5.19	62	4.72	54
No formal qualifications	26.09	161	28.7	343	27.62	316
School‐based qualifications	50.08	309	50.79	607	52.19	597
Tertiary qualification	18.15	112	15.31	183	15.47	177
Ability to manage on available income						
Missing	<1^c^	<5^b^	0.67	8	1.31	15
Easy/not too bad	78.00^c^	480^b^	74.98	896	73.08	836
Difficult/impossible	20.00^c^	120^b^	24.35	291	25.61	293
Number of chronic diseases*						
0 or 1	42.79	264	30.29	362	29.02	332
2 or 3	49.59	306	52.05	622	52.88	605
4 or more	7.62	47	17.66	211	18.09	207

Abbreviation: RAC, residential aged care.

^a^ For the purpose of descriptive frequencies, dementia status is presented as a proportion at the beginning and end of follow‐up; however, in the modelling stage this is treated as a time‐varying covariate.

^b^ These numbers have been slightly changed to avoid low cell sizes (*n* = <5).

^c^ These percentages relate to cells with *n* ≤ 5; in order to maintain anonymity, these percentages are modified slightly and a “<1%” cell is included. They may not add to 100%.

* Statistically significant difference (*P* < 0.05) between event outcomes.

Initial unadjusted models showed that women who had a health assessment were less likely to be admitted to RAC compared with women who did not have a health assessment in the short term. This was true for women with dementia (SDHR_*t* = 100_ = 0.35, 95% CI = [0.21, 0.59]) and for women without dementia (SDHR_*t* = 100_ = 0.39, 95% CI = [0.25, 0.61]). The effect was significant at 100‐days follow‐up, but not at 500‐ or 1000‐days follow‐up. At 2000‐days follow‐up, women who had a health assessment were more likely to be admitted to RAC (Table 2). Women with dementia had a higher likelihood of admission to RAC; however, there was no statistically significant difference between those who did and did not have a health assessment, that is, health assessments were equally effective for women with and without dementia (Table 2).

**Table 2:** Subdistribution hazard ratio (SDHR) estimates for variables of interest in the unadjusted models

		SDHR (95% CI)	SDHR (95% CI)
Parameter	Level	Dementia = No	Dementia = Yes
Health assessment*	Yes vs. No (*t* = 100)	0.39 (0.25, 0.61)	0.35 (0.21, 0.59)
	Yes vs. No (*t* = 500)	0.82 (0.64, 1.05)	0.75 (0.54, 1.03)
	Yes vs. No (*t* = 1000)	1.12 (0.94, 1.35)	1.03 (0.79, 1.33)
	Yes vs. No (*t* = 2000)	1.55 (1.32, 1.82)	1.41 (1.12, 1.79)

		Health assessment = No	Health assessment = Yes
Dementia*	Yes vs. No (*t* = 100)	19.82 (12.95, 30.32)	18.11 (11.43, 28.71)
	Yes vs. No (*t* = 500)	13.73 (10.56, 17.84)	12.54 (9.55, 16.47)
	Yes vs. No (*t* = 1000)	11.72 (9.37, 14.65)	10.71 (8.68, 13.22)
	Yes vs. No (*t* = 2000)	10.01 (8.02, 12.48)	9.14 (7.64, 10.94)

* Variable violated the proportional hazards assumption so SDHRs are presented at 100‐, 500‐, 1000‐ and 2000‐days follow‐up.

The adjusted model showed a small but insignificant reduction in effect magnitude for both health assessment and dementia status as predictors of RAC admission compared with the unadjusted model (Table 3). The same pattern was observed as in the unadjusted model. There was a reduction in likelihood of admission to RAC shortly after health assessments (*t* = 100) and in increase in likelihood of admission to RAC at *t* = 2000 but no significant differences in the intervening period (*t* = 500/1000). Women who were older, living in inner regional areas (vs. major cities), found it difficult/impossible to manage on their available income, and were unpartnered (vs. partnered) had an increased risk of admission to RAC. Women who had high levels of physical functioning were less likely to be admitted to RAC (see Table S1 for SDHR estimates for covariates).

**Table 3:** Subdistribution hazard ratio (SDHR) estimates for variables of interest in the adjusted models

		HR (95% CI)	HR (95% CI)
Parameter	Level	Dementia = No	Dementia = Yes
Health assessment*	Yes vs. No (*t* = 100)	0.40 (0.25, 0.64)	0.38 (0.22, 0.66)
	Yes vs. No (*t* = 500)	0.82 (0.64, 1.06)	0.79 (0.56, 1.12)
	Yes vs. No (*t* = 1000)	1.12 (0.93, 1.35)	1.08 (0.82, 1.43)
	Yes vs. No (*t* = 2000)	2.03 (1.66, 2.47)	1.95 (1.51, 2.52)

		Health assessment = No	Health assessment = Yes
Dementia*	Yes vs. No (*t* = 100)	17.39 (10.96, 27.58)	16.73 (10.18, 27.52)
	Yes vs. No (*t* = 500)	12.80 (9.64, 16.98)	12.32 (9.19, 16.50)
	Yes vs. No (*t* = 1000)	11.21 (8.84, 14.23)	10.79 (8.64, 13.49)
	Yes vs. No (*t* = 2000)	8.74 (6.73, 11.34)	8.41 (6.94, 10.20)

* Variable violated the proportional hazards assumption so SDHRs are presented at 100‐, 500‐, 1000‐ and 2000‐days follow‐up.

## Discussion

The potential for health assessments to improve health outcomes and thereby delay or prevent admission to RAC, at least in the short term, is of tremendous value. In these analyses we demonstrate this potential effect but show that it is time dependent and no longer statistically significant more than 500 days on from the conduct of the health assessment. For those who survive to 2000 days after the assessment, those who have had an assessment are more likely to be admitted to RAC. Many statistically significant covariates were found, including older age, dementia, worse physical functioning, ability to manage on available income, and partnered status, indicating that factors other than health assessments may be more important determinants of admission to aged care than completion of a health assessment.

These findings suggest that health assessments may help delay admission to RAC but may need to be repeated at least every 1–2 years (<500 days) for effects to be sustained. In a review of 21 trials, Beswick et al.^7^ found that geriatric assessments for community‐dwelling older people were marginally protective against nursing home admission (risk ratio [RR] = 0.86 [0.83–0.90]) but with heterogeneity across trial findings. Likewise, Fletcher et al. in a large trial of 106 general practices in the UK did not find a significant reduction in admissions to aged care when comparing universal assessments with targeted assessments.^30^ Against these findings, a randomized controlled trial of home visits conducted in Australia showed an increase in admission to aged care for those in the assessment arm, potentially due to increased assessment and advice about accessing aged care services.^6^

While there is a strong preference for all older people, including people with dementia, to be cared for in the community, current aged care systems can be inadequate to meet the increasingly complex needs of older people and their carers.^31^ The extent to which health assessments could practically prevent RAC admissions among people with higher levels of need is highly dependent on the ability to respond to identified needs. In Australia there is limited integration between health assessments undertaken in general practice (MBS) and assessment for aged care undertaken by the Aged Care Assessment Program.^32^ A general practitioner or nurse may recommend that the older person enroll for aged care assessment, but they can have little impact on the timing or type of services. Other analysis found that earlier admission to home and community care services could delay admission to RAC (amongst women who entered RAC), and better links between general practice assessment of needs and access to services may potentiate this benefit.^33^

While the effects of health assessments on aged care utilization have been debated, with various effects from different trials in different settings,^34^ it may be that other more intensive interventions have more effect, including prevention programs that deliver rehabilitative services within home or community settings.^7^, ^34^

Dementia greatly increases the risk of being admitted to RAC. The effect of dementia as a determinant of RAC admission may overwhelm the potential for assessments to change the course of aged care admission among women with this condition. However, in our data the interaction between dementia and health assessment was not significant in either model. The implication is that there is no evidence to suggest that the effects of health assessments differ for women with and without dementia. Our assessment of dementia in this study is, however, limited, because the women were mostly identified as having dementia in the linked data. Owing to the long time between the beginning of dementia symptoms and a diagnosis,^19^ many women may have had dementia well before this date, and many women with dementia may not have been diagnosed by a doctor or have had this diagnosis recorded in the administrative data. There is also the potential that having a health assessment increased the chances of the person being identified with dementia; however, previous research found no association between these two.^15^

It seems likely that any benefits accruing from health assessments will be dependent on the recency of the assessment (seen here) and repeated assessment (seen in other studies^7^). According to Medicare policy, assessments can occur every year. There remains the potential to improve the conduct of health assessments for older people, and their outcomes. There needs to be greater attention to the uptake of assessments, to repeating and comparing assessments from year to year, to identifying and anticipating needs, and to ensuring adequate follow‐up to meet the needs identified. It is essential to understand barriers to uptake and repeat assessments, and the factors that might limit follow‐up of the needs identified during the assessments. Whether these improvements will better prevent admission to RAC remains to be determined.

## Disclosure statement

The authors declare no conflict of interest.

## Supporting information


**Figure S1.** Selection criteria.

**Table S1.** Covariate subdistribution hazard ratio (SDHR) estimates from the fully adjusted model.

**Table S2.** Maximum standardized differences across imputations for the unmatched and propensity score matched sample.

## Data Availability

ALSWH survey data are owned by the Australian Government Department of Health and due to the personal nature of the data collected, release by ALSWH is subject to strict contractual and ethical restrictions. Ethical review of ALSWH is by the Human Research Ethics Committees at The University of Queensland and The University of Newcastle. De‐identified data are available to collaborating researchers where a formal request to make use of the material has been approved by the ALSWH Data Access Committee. The committee is receptive of requests for datasets required to replicate results. Information on applying for ALSWH data is available from https://alswh.org.au/for-data-users/applying-for-data/.In addition, linked administrative data have been provided by the following third parties [insert list of Data Custodians and HRECs*]. In order for these linked data to be accessed through ALSWH, every data user must be added to the applicable Data Use Agreements and Human Research Ethics Committee protocols.
