# Supplementary figures and images for: Do health assessments affect time to permanent residential aged care admission for older women with and without dementia?
Source: Geriatr Gerontol Int. 2023 Jun 29;23(8):595–602. doi: 10.1111/ggi.14631 (PMC10947059; doi:10.1111/ggi.14631)

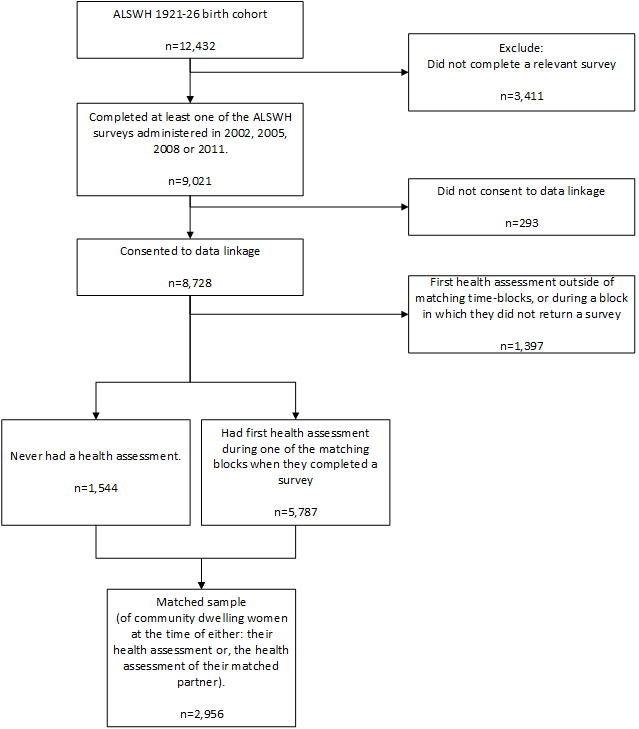

Supplement: Supplementary file 1 — Figure S1. Selection criteria. [file GGI-23-595-s003.png]
