# Supplementary material for: Do health assessments affect time to permanent residential aged care admission for older women with and without dementia?
Source: Geriatr Gerontol Int. 2023 Jun 29;23(8):595–602. doi: 10.1111/ggi.14631 (PMC10947059; doi:10.1111/ggi.14631)
Supplement: Supplementary file 2 — Table S1. Covariate subdistribution hazard ratio (SDHR) estimates from the fully adjusted model. [file GGI-23-595-s001.docx]

Supplementary table 1. Covariate SDHR estimates from the fully adjusted model.

| **Parameter** | **Level** | **SDHR (95% CI)** |
| --- | --- | --- |
| ***Age*** |  | 1.12 (1.07, 1.16) |
| ***Number of chronic conditions*** | *0 or 1* | ref |
|  | *2 or 3* | 1.09 (0.93, 1.28) |
|  | *4 or more* | 1.13 (0.89, 1.43) |
| ***Concession card*** | *No* | ref |
|  | *Yes* | 1.02 (0.86, 1.20) |
| ***Fall in the last 12 months*** | *No* | ref |
|  | *Yes* | 1.13 (0.96, 1.34) |
| ***Self-rated general health*** | *Excellent/Very good/good* | ref |
|  | *Fair/Poor* | 0.88 (0.70, 1.10) |
| ***SF36 general health subscale score*** |  | 1.00 (0.99, 1.00) |
| ***Number of GP visits in previous 12 months*** |  | 1.00 (0.99, 1.01) |
| ***SF36 mental health subscale score*** |  | 1.00 (1.00, 1.01) |
| ***Highest Qualification*** | *No formal qualifications* | ref |
|  | *School based qualifications* | 1.16 (0.98, 1.37) |
|  | *Tertiary qualification* | 1.14 (0.92, 1.42) |
| ***Area of residence*** | *Major city* | ref |
|  | *Inner regional* | 1.20 (1.03, 1.40) |
|  | *Outer regional/Rural/Remote* | 0.96 (0.80, 1.15) |
| ***Ability to manage on available income**** | *Difficult/impossible vs Easy/not too bad (t=100)* | 1.85 (1.16, 2.95) |
|  | *Difficult/impossible vs Easy/not too bad (t=500)* | 1.36 (1.06, 1.75) |
|  | *Difficult/impossible vs Easy/not too bad (t=1000)* | 1.20 (1.00, 1.44) |
|  | *Difficult/impossible vs Easy/not too bad (t=2000)* | 0.93 (0.77, 1.14) |
| ***SF 36 Physical functioning subscale score**** | *(t=100)* | 0.97 (0.97, 0.98) |
|  | *(t=500)* | 0.99 (0.98, 0.99) |
|  | *(t=1000)* | 0.99 (0.99, 1.00) |
|  | *(t=2000)* | 1.00 (1.00, 1.01) |
| ***Partnered status**** | *Unpartnered vs Partnered (t=100)* | 1.91 (1.15, 3.20) |
|  | *Unpartnered vs Partnered (t=500)* | 1.44 (1.10, 1.90) |
|  | *Unpartnered vs Partnered (t=1000)* | 1.28 (1.06, 1.54) |
|  | *Unpartnered vs Partnered (t=2000)* | 1.02 (0.85, 1.21) |

**NOTE: These are covariates in the final model that also contains dementia, health assessment, their interaction, and their log(time) interaction. The estimates for these variables are presented in table 3.**

*NOTE: variable violated the proportional hazards assumption so SDHRs are presented at 100-, 500-, 1000- and 2000-days follow-up
