# Supplementary material for: Do health assessments affect time to permanent residential aged care admission for older women with and without dementia?
Source: Geriatr Gerontol Int. 2023 Jun 29;23(8):595–602. doi: 10.1111/ggi.14631 (PMC10947059; doi:10.1111/ggi.14631)
Supplement: Supplementary file 3 — Table S2. Maximum standardized differences across imputations for the unmatched and propensity score matched sample. [file GGI-23-595-s002.docx]

Supplementary table 2. Maximum standardised differences across imputations for the unmatched and propensity score matched sample.

|  | **Maximum absolute standardised differences across imputations** | | | | |
| --- | --- | --- | --- | --- | --- |
|  | **Unmatched Sample*** | | | | **PS - Matched Sample** |
| **Characteristic** | *Block 1* | *Block 2* | *Block 3* | *Block 4* |  |
| *Sample - size (n)* | 5,235 | 2,150 | 1,238 | 679 | 2,956 |
| *Age* | 0.05 | 0.11 | 0.08 | 0.05 | 0.01 |
| *SF36 mental health subscale score* | 0.01 | 0.09 | 0.12 | 0.18 | 0.03 |
| *SF36 general health subscale score* | 0.18 | 0.15 | 0.04 | 0.07 | 0.04 |
| *SF36 physical functioning subscale score* | 0.16 | 0.10 | 0.04 | 0.05 | 0.02 |
| *Number of GP attendances in the previous year* | 0.15 | 0.19 | 0.19 | 0.28 | 0.04 |
| *Dementia (at end of matching block)* | 0.10 | 0.04 | 0.16 | 0.18 | 0.03 |
| *Fall to the ground in the last 12 months* | 0.09 | 0.03 | 0.09 | 0.07 | 0.04 |
| *Concession card status* | 0.18 | 0.15 | 0.15 | 0.01 | 0.05 |
| *Self-reported general health* | 0.15 | 0.11 | 0.01 | 0.14 | 0.04 |
| *Area of residence* | 0.06 | 0.13 | 0.26 | 0.07 | 0.02 |
| *Partnered status* | 0.10 | 0.08 | 0.15 | 0.07 | 0.03 |
| *Highest qualification* | 0.09 | 0.11 | 0.09 | 0.10 | 0.03 |
| *Ability to manage on available income* | 0.03 | 0.11 | 0.05 | 0.03 | 0.01 |
| *Number of chronic diseases* | 0.02 | 0.00 | 0.05 | 0.06 | 0.03 |
